# Gut microbiota composition does not associate with *toxoplasma* infection in rats

**DOI:** 10.1111/mec.16552

**Published:** 2022-06-12

**Authors:** Patrick L. Taggart, Craig Liddicoat, Wen Han Tong, Martin F. Breed, Philip Weinstein, David Wheeler, Ajai Vyas

**Affiliations:** ^1^ School of Animal and Veterinary Sciences The University of Adelaide Roseworthy South Australia Australia; ^2^ Vertebrate Pest Research Unit, Department of Primary Industries NSW Queanbeyan New South Wales Australia; ^3^ School of Biological, Earth and Environmental Sciences University of New South Wales Sydney New South Wales Australia; ^4^ College of Science and Engineering Flinders University Bedford Park South Australia Australia; ^5^ School of Public Health The University of Adelaide Adelaide South Australia Australia; ^6^ School of Biological Sciences Nanyang Technological University (SBS‐NTU) Singapore Singapore; ^7^ Chief Scientist Unit Department of Primary Industries NSW Orange New South Wales Australia

**Keywords:** behavioural manipulation, inflammation, microbiome, microbiota, parasite, *toxoplasma*

## Abstract

*Toxoplasma* infection in intermediate host species closely associates with inflammation. This association has led to suggestions that the behavioural changes associated with infection may be indirectly driven by the resulting sustained inflammation rather than a direct behavioural manipulation by the parasite. If this is correct, sustained inflammation in chronically infected rodents should present as widespread differences in the gastrointestinal microbiota due to the dependency between the composition of these microbiota and sustained inflammation. We conducted a randomized controlled experiment in rats that were assigned to a *Toxoplasma*‐treatment, placebo‐treatment or negative control group. We euthanised rats during the chronic phase of infection, collected their caecal stool samples and sequenced the V3‐V4 region of the 16S rRNA gene to characterize the bacterial community in these samples. *Toxoplasma* infection did not induce widespread differences in the bacterial community composition of the gastrointestinal tract of rats. Rather, we found sex differences in the bacterial community composition of rats. We conclude that it is unlikely that sustained inflammation is the mechanism driving the highly specific behavioural changes observed in *Toxoplasma*‐positive rats.

## 1 INTRODUCTION

Laboratory rodents retain an aversion to feline odours (Dielenberg & McGregor, 2001). This aversion does not require prior learning and can drive conditioned learning by itself (Takahashi et al., 2008). The absence of prior learning suggests that this aversion to feline odours by rodents is an innate behaviour and is not removed through a long history of laboratory domestication in the absence of feline predators. However, this innate aversion to feline odours is reduced in laboratory rodents when they are chronically infected with the protozoan parasite *Toxoplasma gondii* (Berdoy et al., 2000).

Infection with *Toxoplasma* induces an acute phase of inflammation, followed by a less tumultuous chronic phase characterized by the parasite's encystment within immune‐privileged organs of the body (Jeffers et al., 2018). When consumed by felines, the parasite penetrates and infects through the gut wall, where it undergoes gametogenesis, and later emerges as a copious inoculum of environmentally stable oocysts in the faeces (Dubey, 2009). Host behavioural manipulation by *Toxoplasma* potentially explains the loss of fear in infected rodents, as the process of parasite gametogenesis requires the predation of the infected rodent and is assisted by their reduced fear towards cats (Abdulai‐Saiku et al., 2021). This view emphasizes a direct relationship between the parasitism and the host changes, possibly an adaptive change that increases parasite transmission (Poulin, 1995).

An alternative explanation to the loss of fear in infected rodents is that it is one component of widespread changes in their behaviour as a result of inflammation induced by *Toxoplasma* infection; a response analogous to a generalized alteration in behaviour without a specific discernable stimuli (Boillat et al., 2020; Tyebji et al., 2020). Thus, any ongoing inflammation during the chronic phase of *Toxoplasma* infection, caused by the parasite's periodic recrudescence, or lasting effects of acute inflammation early in the infection, could potentially cause a range of behavioural changes including the loss of innate aversion (Boillat et al., 2020). In this context, host behavioural changes are the result of indirect and nonspecific changes due to inflammation rather than a specific manipulation of rodent behaviour by the parasite. This role of inflammation would be supported if *Toxoplasma* infection causes changes in rodent phenotypes known to be sensitive to long‐term inflammation, for example, the gastrointestinal microbiota.

The gastrointestinal microbiota is one of the main components of the body affected by ongoing inflammation. For example, mice “enterotypes” with low microbial richness coexist with higher inflammation, even when overt signs of sickness are absent, suggesting that low‐grade and continual inflammation is associated with microbiota dysbiosis (Hildebrand et al., 2013). Similarly, indomethacin, a nonsteroidal anti‐inflammatory drug, alters the intestinal microbiota of mice when administered either acutely or chronically, suggesting that inflammation is causally linked to microbial composition in rodents (Liang et al., 2015). The use of these anti‐inflammatory drugs also results in compositional changes in the human gastrointestinal microbiota (Rogers & Aronoff, 2016). Again, the reduction of interleukin activity in a mouse model of intestinal inflammatory disease also causes a taxonomic shift in the gastrointestinal microbiota (Menghini et al., 2019). The exogenous administration of dexamethasone over several weeks, an analogue of adrenal glucocorticoids that suppresses inflammation, also results in changes in the composition of the gastrointestinal microbiota in mice (Huang et al., 2015). Thus, long‐term inflammation can shape the community of microbiota in the gut (Huang et al., 2015).

This reciprocal relationship between inflammation and the gastrointestinal microbiota is thought to influence a variety of animal phenotypes, including host physiology and behaviour (Spor et al., 2011). Accordingly, the inflammatory landscape induced by *Toxoplasma* infection is expected to alter the composition of microbes observed in the gastrointestinal tract. Here, we tested this hypothesis by undertaking a randomized controlled experiment of *Toxoplasma* infected rats (*Rattus norvegicus*) and examining the effect of infection on the composition of the gastrointestinal microbiota.

## 2 MATERIALS AND METHODS

### 2.1 Infection of laboratory rats

We obtained 50 eight‐week old male and female Wistar Hannover rats (HanTac:WH; 24 male rats, 24 female rats and 2 control rats) from InVivos Private Limited, Singapore. All rats were housed in pairs with ad libitum supply of food and water (12:12 h light–dark cycle, lights on at 07:00 AM). Rats were allowed to acclimatize to the vivarium for 1 week prior to the start of experiments. We randomly assigned rodent cages to two experimental groups based on blinded inoculums. Rats assigned to the treatment group received five million *Toxoplasma gondii* tachyzoites in 500 μl buffered saline via the intraperitoneal route. Those assigned to the control group received only saline via the same route. The intraperitoneal route was chosen to maintain consistency with earlier studies, and to variabilities known to occur with the use of oral gavage (Boyle et al., 2007). *Toxoplasma* parasites were maintained in monolayers of immortalized human foreskin fibroblasts (ATCC no. CRL‐4001) and harvested through syringe lysis with a 25‐gauge needle, as previously described (Tan et al., 2020; Vyas, Kim, Giacomini, et al., 2007). A type II Prugniaud (Pru) *Toxoplasma gondii* strain was used in the experiments. We used two of the 50 rodents as negative controls (i.e., rats were not inoculated with anything but instead used to generate sampling blank controls). Both negative control rats were subject to equivalent experimental conditions for the duration of our laboratory experiment and sample processing. All animal experimental procedures were conducted at the Nanyang Technological University and reviewed and approved by the institutional Animal Care and Use Committee (no. A18074).

### 2.2 DNA extraction, PCR and sequencing

All rodents were euthanised at eight weeks post‐infection to ensure they had reached the chronic phase of the *Toxoplasma gondii* infection (Dubey et al., 1997). Rodents were then presented on sterile boards and their fur sprayed with ethanol to minimize contamination of internal tissues. Rodent caeca were then harvested under a flow hood, snap‐frozen in liquid nitrogen, and stored at –80°C. Total DNA was extracted from 250 mg of stool collected from within each cecum sample using the QIAamp PowerFecal Pro DNA Kit (Qiagen no. 51804), following instructions from the manufacture. DNA samples were then stored at –80°C prior to shipping to Adelaide, Australia, on dry ice. The experimenter was blind to the infection status of all rodents during the isolation of DNA, and the samples were processed in a random order.

DNA amplification was performed at the Australian Genome Research facility (AGRF). AGRF amplified the bacterial 16S rRNA V3‐V4 gene region using forward primer 341F (5′‐ CCTAYGGGRBGCASCAG‐ 3′) and reverse primer 806R (GGACTACNNGGGTATCTAAT). A modified Illumina two‐stage PCR library preparation technique was then used, adapted from (Illumina, 2014). Primary PCR amplicons were generated using region‐specific primers coupled with nextera stubby forward (5’‐TCGTCGGCAGCGTCAGATGTGTATAAGAGACAG‐ 3′) and reverse (5’‐GTCTCGTGGGCTCGGAGATGTGTATAAGAGACAG‐ 3′) adapter sequences and Platinum SuperFi (ThermoFisher). PCR reactions were cycled at 95°C for 7 min; 29 cycles of 94°C for 30 s, 50°C for 60 s and 72°C for 60 s; and final extension at 72°C for 7 min. The first stage PCR was cleaned using a 1.8× ratio of Sera‐Mag Magnetic Speedbeads (Merck), and samples were visualized on 2% SYBR E‐Gel (Thermo‐Fisher). A secondary PCR was performed with PrimeSTAR Max DNA polymerase (Takara) to add sample indices and Illumina P5/P7 sequencing adaptors to the first stage amplicons using unique‐dual indexing. Second stage PCR reactions were cycled at 98°C for 10 s; eight cycles of 98°C for 10 s, 50°C for 5 s and 72°C for 10 s, and holding at 10°C. The resulting amplicons were cleaned again using a 1.8× ratio of magnetic beads, quantified by fluorometry (Promega QuantiFluor) and then normalized. The eqimolar pool was cleaned a final time using a 1.5× ratio of magnetic beads to concentrate the pool and then measured using a high‐sensitivity D1000 TapeStation assay (Agilent). The pool was diluted to 5 nM and molarity was confirmed again using a HighSensitivity D1000 TapeStation assay (Agilent, USA).

Sequencing was performed at the AGRF using a MiSeq with V3 600 cycle chemistry (300 base‐pair paired‐end), 25% PhiX and clustered at 7 pM.

### 2.3 Sequence analysis

Amplicon sequence variants (ASVs) were generated using the QIIME2 (version r2021.11) analysis pipeline (https://doi.org/10.1038/s41587‐019‐0209‐9). Paired end 300 bp Illumina reads were first trimmed on primers using the cutadapt trim‐paired QIIME2 plugin with the default parameters. Sequences were denoised, overlapped, dereplicated, and filtered for chimeras using the DADA2 QIIME2 plugin, with the following parameters: ‐‐p‐trunc‐len‐f 240, −‐p‐trunc‐len‐r 220, −‐p‐max‐ee‐f 2, −‐p‐max‐ee‐r 4, −‐p‐n‐reads‐learn 1,000,000, and ‐‐p‐chimera‐method consensus (Martin, 2011). For classifier training the 341F and 806R amplicon region was extracted from the QIIME compatible SILVA SSU database release 138 using the qiime feature‐classifier extract‐reads command (Quast et al., 2012). The classifier model was then fit and trained using the commands qiime feature‐classifier fit‐classifier‐native‐bayes and qiime feature‐classifier classify‐sklearn with the default parameters. Run scripts and conda env files used in this analysis can be found at the projects git repository https://bitbucket.org/dpi_data_analytics/202202taggart/src/master/.

### 2.4 Data cleaning

We discarded ASVs that were not identified as bacteria, were unidentified at the phylum level, assigned as chloroplast or mitochondria, or did not occur in at least two samples. We identified and removed contaminating sequences using the decontam package in R via the isContaminant() function as recommended in the decontam user guide for high biomass sample types (Davis et al., 2018; R Core Team, 2020). We used the prevalence method which identifies contaminants by increased presence in negative controls. Otherwise, default settings were used throughout. Subsequently, all taxa identified as contaminants and the negative control samples were removed from further microbiota data analysis. After these data cleaning steps, a total of 829 ASVs from 48 samples (2,474,934 total sequences) remained.

### 2.5 Statistical analysis

We used R software and the microbiome data analysis framework of the phyloseq package (McMurdie & Holmes, 2013; R Core Team, 2020). Based on rarefaction curve plots two samples with very low sequence counts (5AB/11BF) were discarded and ASV abundance data were rarefied to the minimum sample sequence read depth of the remaining samples (17,577 sequences) (Figure S1). We discovered two outlier samples in beta diversity analyses and so for these analyses we discarded two additional samples (2BF/7BF) giving a rarefaction depth of 18,165 sequences. We estimated alpha diversity in each sample via the exponential transform of Shannon index values to derive the effective number of ASVs (Jost, 2006). These data were visualized using boxplots, a heat map and barplot grouped by treatment and gender. We tested for differences in alpha diversity between the groups using the Kruskal‐Wallis rank sum test in base R (version 4.1.2), followed by the Dunn test for multiple comparisons to determine which groups differed from other groups, with Bonferroni‐adjusted threshold *p*‐values. The multiple comparison testing used default two‐sided *p*‐values and an alpha = 0.05 nominal level of significance.

We visualized differences between sample microbiota (beta diversity) using non‐metric multidimensional scaling (NMDS) ordination of Bray‐Curtis distances. Two outliers samples, 2BF (female placebo) and 7BF (female toxoplasma), were excluded from the beta diversity analysis due to outlying high NMDS1 and NMDS2 scores ‐ that is, microbiota data with the outlier removed (total 829 ASVs; 44 samples; 2,377,578 sequences) was then rarefied again to the lowest sample depth (18,165 sequences) for this beta diversity analysis. We used permutational multivariate analysis of variance (PERMANOVA), as implemented in the adonis() function within the vegan package, to test for compositional differences by treatment and sex between microbiota sample groups (Oksanen et al., 2020). This was followed by testing for homogeneity of group dispersions via the betadisper() function (also from *vegan*).

We also assessed differentially abundant ASVs (again using microbiota data with the two outlier samples 2BF and 7BF removed) between the *Toxoplasma* versus placebo treatments using the ANCOM‐BC package (version 1.4; 10.1038/s41467‐020‐17,041‐7). Differential ASV abundance was calculated for both treatment and sex using the *ancombc* function with the default options, except formula = Treatment + Sex, group = Treatment, lib_cut = 1000, neg_lb = TRUE, and conserve = TRUE. *p*‐values were adjusted for multiple comparisons using the default Holm‐Bonferroni method and < = .05 was used as a significance cutoff (Holm, 1979).

The citation for the data related to this article is [data set] Taggart et al. (2022).

## 3 RESULTS

**FIGURE 1:**
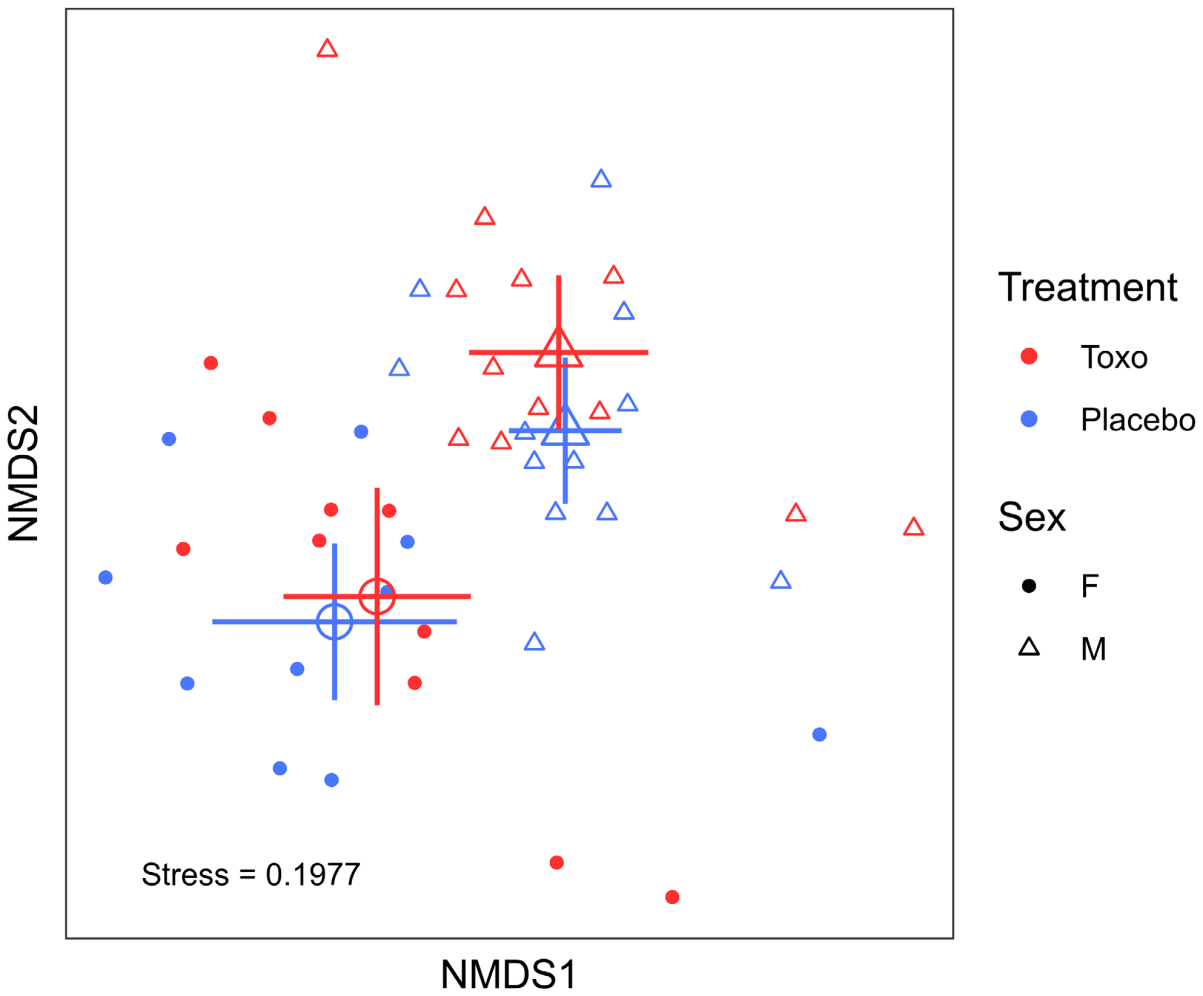
Nonmetric multidimensional scaling (NMDS) plot of bray‐Curtis distances of the rat caecal bacterial communities. The enlarged symbols represent centroids of the respective treatment‐sex groups which have overlapping error bars, each representing 95% confidence intervals (or ± 1.96 × standard error of the mean). Note each point represents the entire bacterial community of a single caecal sample, where closer points are more similar in composition. Shapes denote sex and colour represents treatments for each sample. All samples have been rarefied to 18,165 sequences and two samples (2BF/7BF) have been excluded due to an outlying high NMDS1 and NMDS2 scores. Samples represent 12 male and 10 female toxoplasma treatment rats and 12 male and 10 female placebo treatment rats

Following sample data cleaning, the median total sequences per sample in the *Toxoplasma* and placebo treatment groups was 53,675 (range: 18,165, 71,228) and 57,699 (range: 19,523, 99,413) sequences, respectively (Tables S1 and S2). Female and male rats varied similarly in their total sequences per sample post data cleaning, with a median total sequences per sample among females and males of 51,741 (range: 18,165, 79,103) and 57,951 (range: 18,774, 99,413) sequences, respectively (Tables S1 and S2). Following the removal of two low count samples, the minimum sequence depth for alpha diversity analyses was 17,577 sequences, an additional two outlier samples were removed based on NMDS plots, giving a minimum sequence depth for beta diversity analyses of 18,165 sequences (Figure 1). Both of these rarefaction depths were very near the plateau of diversity in rarefaction curves for each sample (Figure S1).

We found no effect of the *Toxoplasma* versus placebo treatment (PERMANOVA *df* = 1, *F* = 0.91, *R* ^2^ = .021, *p* = .54, *n* = 22 *Toxoplasma* vs. 22 placebo), but a significant effect of sex (PERMANOVA *df* = 1, *F* = 4.62, *R* ^2^ = .099, *p* = .001, *n* = 2 male vs. 20 female), on bacterial community composition (or beta diversity). This was clearly indicated by the nonmetric multidimensional scaling (NMDS) plot of Bray‐Curtis distances of rat caecal bacterial communities (Figure 1). *Toxoplasma* versus placebo treatment rats showed extensive mixing and clear overlapping of treatment groups; in contrast male and female rats showed little mixing and clear separation of the sex groups (Figure 1). Beta‐dispersions did not vary by treatment (*df* = 1, *F* = 0.18, *p* = .66) or sex (*df* = 1, *F* = 0.13, *p* = .71). Again, this was clearly indicated in the NMDS plot by the comparable spread of samples by both treatment and sex (Figure 1).

**FIGURE 2:**
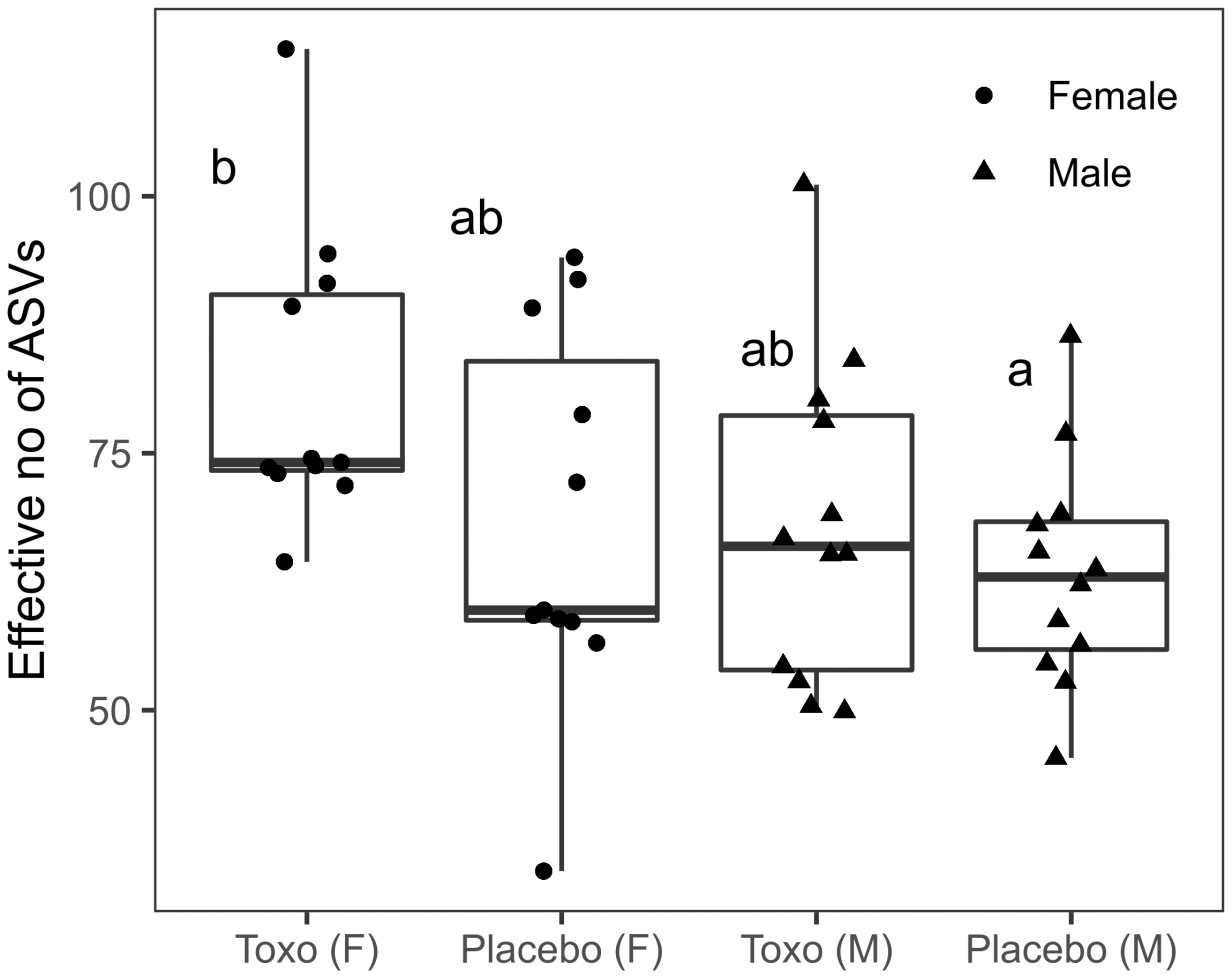
Boxplots of the number of amplicon sequence variants (ASVs). Individual samples are overlayed on the boxplots separated by treatment and sex groupings. The number of rats in each group is 10 (Toxo [F]), 10 (placebo [F]), 12 (Toxo [M]), 12 (placebo [M]). Groups not sharing a letter are significantly different (from post hoc multiple comparison Dunn tests). All samples have been rarefied to 17,577 sequences

**FIGURE 3:**
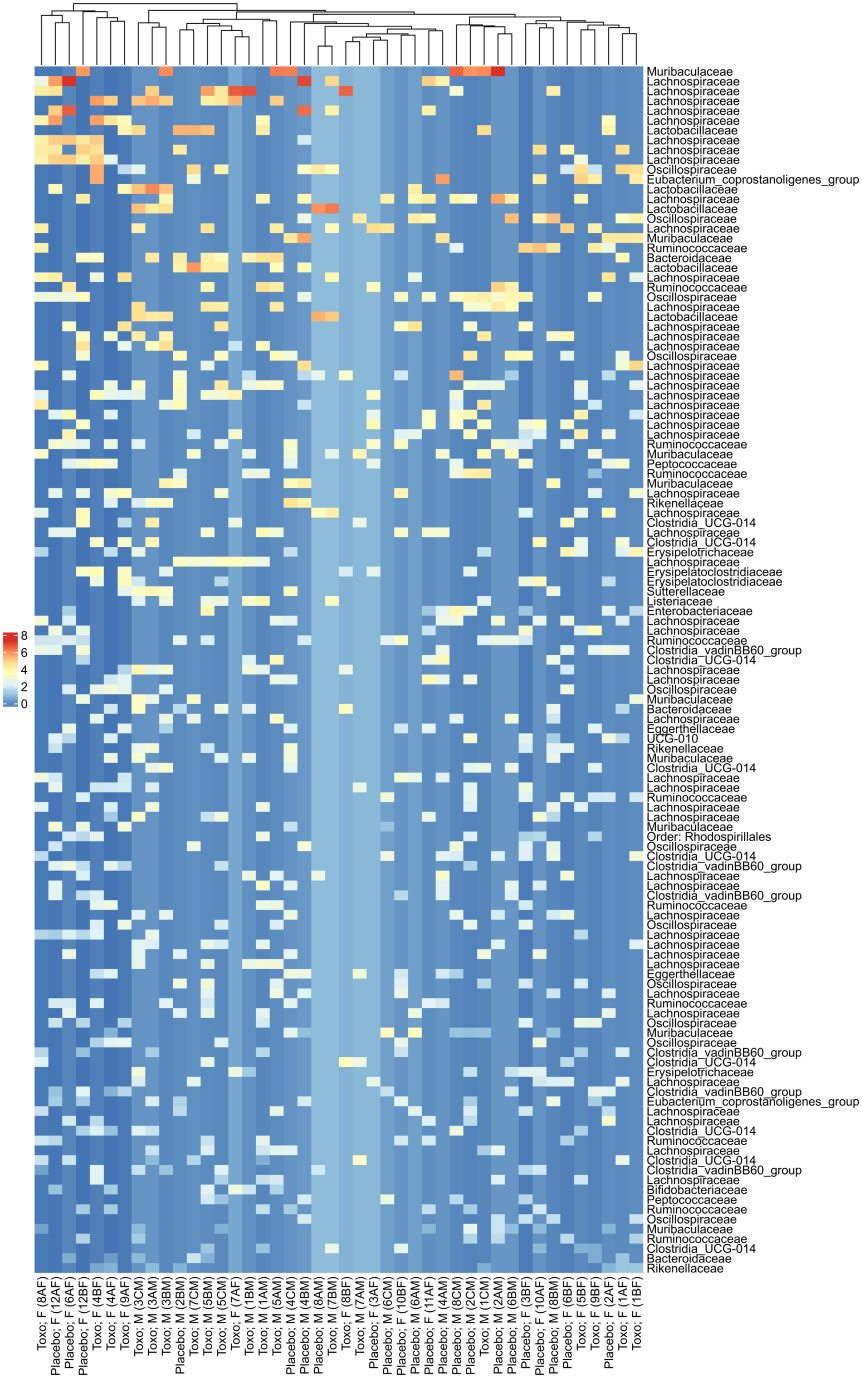
Heatmap depicting bias‐adjusted log observed abundances of taxa identified as significantly different between placebo and toxoplasma treated rats. A positive log value indicates higher abundance in the toxoplasma treated rats, and vice versa. The dendrogram shows clustering based on euclidean distances between samples. Taxa are labelled at the family level, or next available taxonomic classification. All samples have been rarefied to 17,577 sequences

Neither treatment (*Toxoplasma* vs. placebo) nor sex had a significant effect on the effective number of ASVs (exponential of Shannon diversity index; Alpha diversity) (Figure 2). This lack of differences in within‐sample diversity was further illustrated by the heatmap of significantly different bias‐adjusted log observed abundances (Figure 3) and relative abundance barplot (Figure S2), which both show a consistent bacterial composition within all samples.

Although we did not find any major differences in either alpha or beta diversity between treatments, a number of differentially abundant ASVs were identified, including Lachnospiraceae, Oscillospiraceae, Ruminococcaceae, Muribaculaceae and Clostridia UCG‐014 (Table S3). However, the average log fold change across all ASVs within each of these families was <1, and <0.5 for the vast majority of families.

## 4 DISCUSSION

*Toxoplasma* infection in intermediate host species is known to be closely associated with inflammation. This inflammation is most severe during the acute phase of infection but persists during chronic infection due to the parasites' recrudescence (Jeffers et al., 2018). This has led to suggestions that the behavioural changes known to be associated with *Toxoplasma* infection may be driven indirectly by the resulting inflammation, and not the result of a behavioural manipulation by the parasite (Boillat et al., 2020; Tyebji et al., 2020). If this is true, we expected to observe widespread differences in the gastrointestinal microbiota of chronically infected rats as a consequence of the close relationship between infection, sustained inflammation and the gut microbiota composition (Hildebrand et al., 2013; Huang et al., 2015; Liang et al., 2015; Menghini et al., 2019).

However, we found no evidence that *Toxoplasma* infection induced widespread differences in the overall bacterial community composition of the gastrointestinal tract of rats. This suggests that *Toxoplasma* infection may not cause sustained inflammation in chronically infected rats and that behavioural changes known to be associated with *Toxoplasma* infection in rats are unlikely to be driven by sustained inflammation.

Our results contrast with those of Shao et al. (2020) and Prandovszky et al. (2018), who reported substantial changes in the overall bacterial community composition in the gastrointestinal tract of mice chronically infected with *Toxoplasma*. These differences in results may be driven by the differing impacts of *Toxoplasma* infection in mice compared to rats and the implications infection has for sustained inflammation in each species.

In rats, acute *Toxoplasma* infection induces minimal inflammation, sickness behaviours or mortality but highly specific behavioural changes during chronic *Toxoplasma* infection (Berdoy et al., 1995, 2000; Vyas, Kim, Giacomini, et al., 2007; Vyas, Kim, & Sapolsky, 2007). This is in contrast to mice, which experience vigorous inflammation, regularly display sickness behaviours and lose bodyweight during acute *Toxoplasma* infection. Perhaps unsurprisingly, mice also show a lack of, or less specific, behavioural changes during chronic *Toxoplasma* infection (Boillat et al., 2020; Hodková et al., 2007; Hrdá et al., 2000; Soh et al., 2013; Tyebji et al., 2020). Such generalized behavioural changes in mice are typical of inflammation‐induced changes. Inflammation may therefore be involved in behavioural changes associated with *Toxoplasma* infection in mice, but is unlikely to play an important role in the highly specific behavioural changes observed in rats (Abdulai‐Saiku et al., 2021). It is important to note that we examine the possibility of long‐term microbiota reorganization in response to infection‐induced inflammation, yet we do not directly quantify host inflammation in this article.

We show sex differences in the overall bacterial community composition of the gastrointestinal tract of rats. Our results are consistent with sex differences in the microbiota of adult mice (Org et al., 2016; Yurkovetskiy et al., 2013). These differences are known to be driven by gonadal hormones and develop post‐puberty (Org et al., 2016; Yurkovetskiy et al., 2013). Grafting of the male caecal microbiota in female mice is also known to increase testosterone (Markle et al., 2013). It would therefore appear that the gastrointestinal microbiota causes sex differences, in addition to being caused by sex‐specific differences.

Our study design and sample size were sufficient to detect between‐group differences in the overall bacterial community composition of the gastrointestinal tract of rats, when they were present. This is demonstrated by the sex differences we report. Accordingly, when comparing the bacteria community composition of *Toxoplasma* and placebo rats, we had sufficient power to detect differences equal to or greater in size than those observed between sexes. We cannot rule out the possibility that our study had insufficient power to detect small differences in the overall bacterial community composition between *Toxoplasma* and placebo rats. However, sustained inflammation is known to cause widespread changes in the gastrointestinal microbiota of rodents (Hildebrand et al., 2013; Huang et al., 2015; Liang et al., 2015; Menghini et al., 2019). Hence, small changes in the overall bacterial community composition between *Toxoplasma* and placebo rats would nonetheless be inconsistent with the expected results under the sustained inflammation hypothesis. Combined this suggests that behavioural changes in rats associated with *Toxoplasma* infection are unlikely to be driven by sustained inflammation due to infection.

## AUTHOR CONTRIBUTIONS

Patrick L. Taggart, Craig Liddicoat and Philip Weinstein conceived of the study. Ajai Vyas and Wen Han Tong completed laboratory infection trials in rats, collected celal stool samples and extracted microbial DNA. Patrick L. Taggart, Martin F. Breed and Philip Weinstein facilitated secquencing of microbial DNA. Craig Liddicoat and David Wheeler conducted sequence processing and statistical analysis. Patrick L. Taggart, Craig Liddicoat and Ajai Vyas wrote the initial draft of manuscript. All authors contributed to revision and approval of the final manuscript.

## CONFLICT OF INTEREST

The authors declare no competing interests.

### OPEN RESEARCH BADGES

This article has earned an Open Data Badge for making publicly available the digitally‐shareable data necessary to reproduce the reported results. The data is available at https://doi.org/10.5061/dryad.c866t1g7z.

## Supporting information


Fig S1‐S2
Table S1‐S3

## Data Availability

All raw data have been stored on Dryad (doi: 10.5061/dryad.c866t1g7z). R scripts and conda env files used in this analysis can be found at the projects git repository https://bitbucket.org/dpi_data_analytics/202202taggart/src/master/.
