# Supplementary material for: Gut microbiota composition does not associate with toxoplasma infection in rats
Source: Mol Ecol. 2022 Jun 12;31(14):3963–70. doi: 10.1111/mec.16552 (PMC9546062; doi:10.1111/mec.16552)
Supplement: Supplementary file 1 — Fig S1‐S2 Table S1‐S3 [file MEC-31-3963-s001.docx]

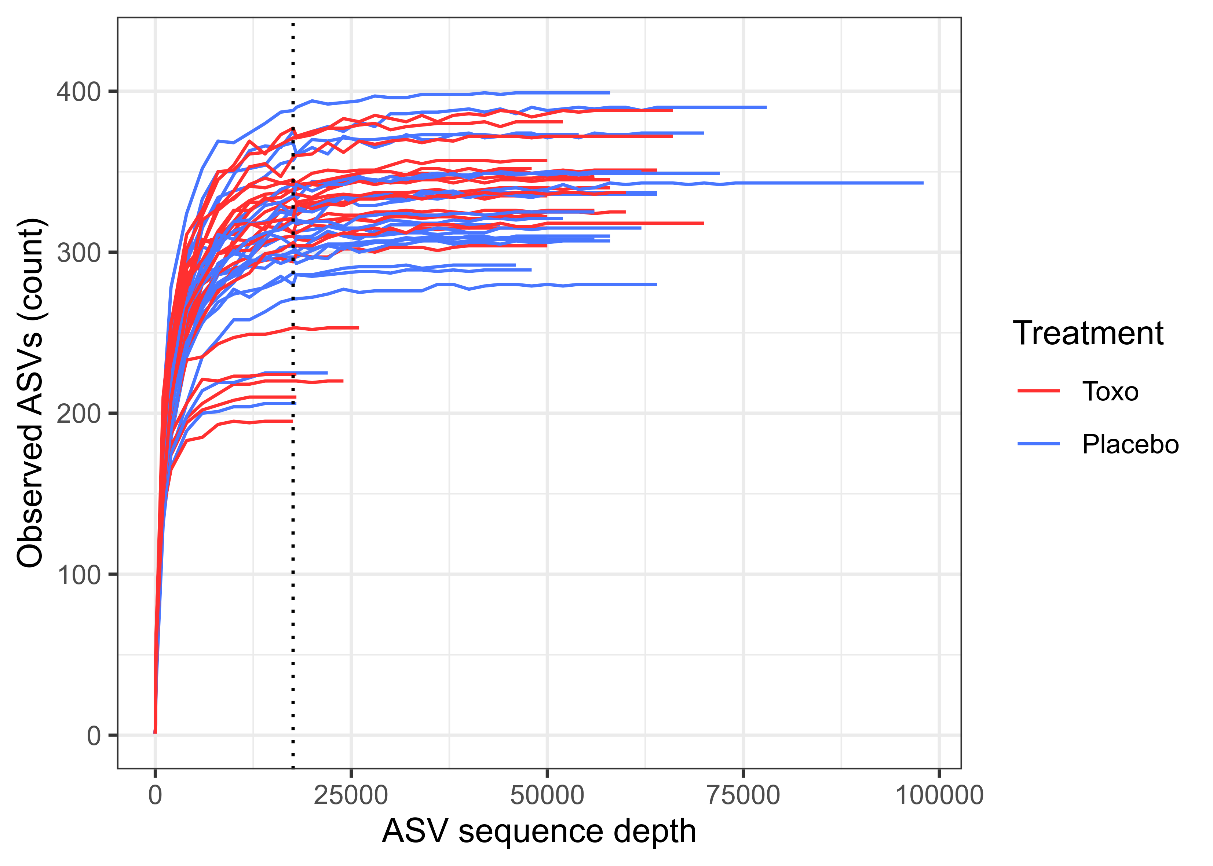


Figure S1: Rarefaction curves for study samples based on cleaned data.

*The vertical dotted line at the value of* 17,577 *sequences corresponds to the minimum sum of sequences within a particular sample after the removal of the two samples with low sequence counts (5AB/11BF).*


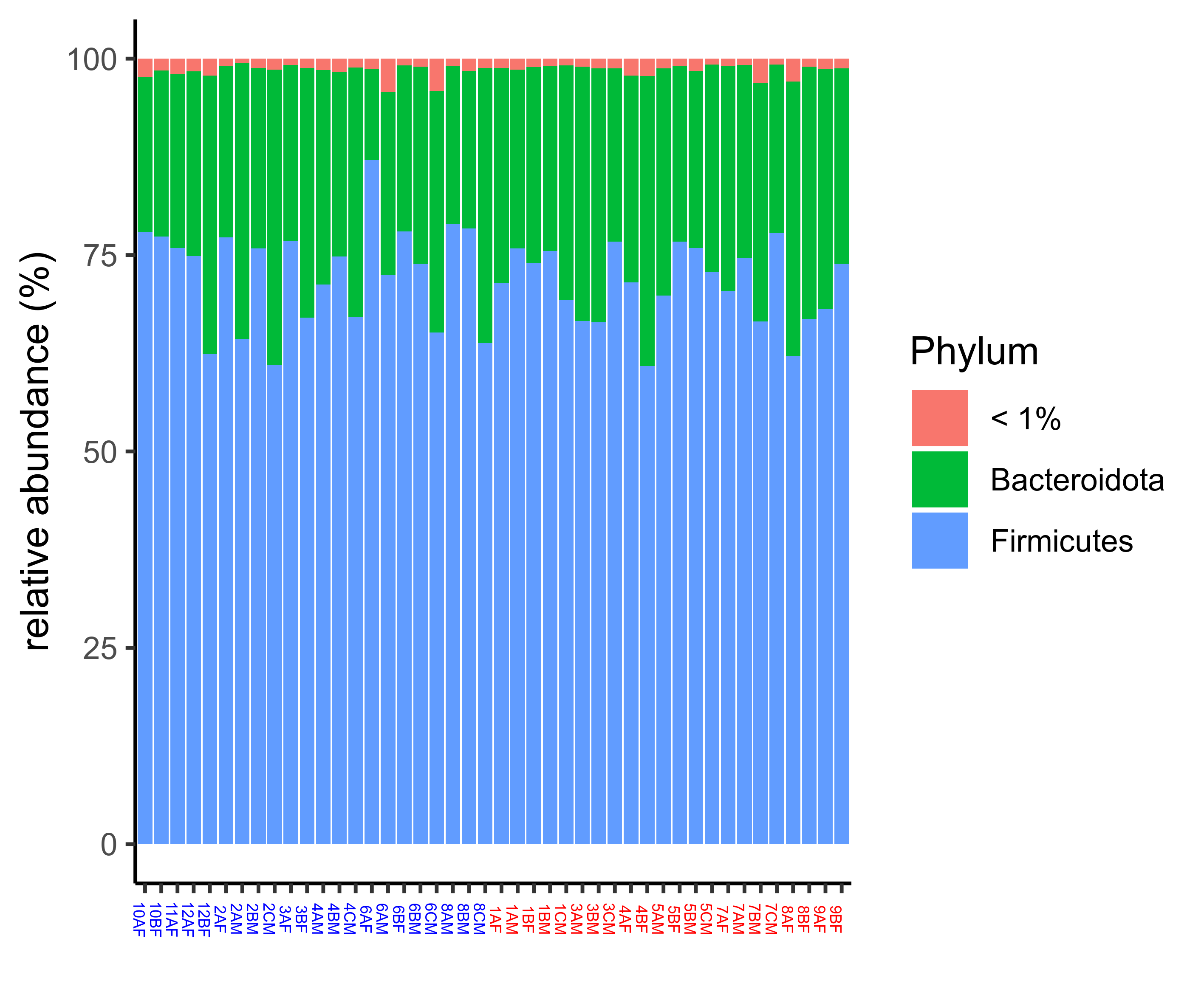


Figure S2: Relative abundance bar plot of major phyla in the study samples.

*The sample labels on the x-axis are coloured by treatment group, with placebo sample in blue and toxoplasma in red. Phyla that were present at <1% included Actinobacteria, Cyanobacteria, Deferribacteres, Proteobacteria, Verrucomicrobiota, Desulfobacterota and Patescibacteria.*

Table S1: Overview of sample information including sequence sums following data cleaning steps (removal of low count samples and outliers based on NMDS ordination plots).

| **Sample ID** | **Sex** | **Treatment** | **Total sequences per sample** |
| --- | --- | --- | --- |
| 1AF | F | *Toxoplasma* | 50914 |
| 1BF | F | *Toxoplasma* | 50734 |
| 4AF | F | *Toxoplasma* | 66020 |
| 4BF | F | *Toxoplasma* | 48621 |
| 5BF | F | *Toxoplasma* | 50513 |
| 7AF | F | *Toxoplasma* | 27859 |
| 8AF | F | *Toxoplasma* | 18165 |
| 8BF | F | *Toxoplasma* | 66581 |
| 9AF | F | *Toxoplasma* | 60108 |
| 9BF | F | *Toxoplasma* | 57626 |
| 1AM | M | *Toxoplasma* | 57443 |
| 1BM | M | *Toxoplasma* | 64593 |
| 1CM | M | *Toxoplasma* | 51376 |
| 3AM | M | *Toxoplasma* | 59905 |
| 3BM | M | *Toxoplasma* | 60273 |
| 3CM | M | *Toxoplasma* | 55197 |
| 5AM | M | *Toxoplasma* | 58487 |
| 5BM | M | *Toxoplasma* | 51579 |
| 5CM | M | *Toxoplasma* | 24339 |
| 7AM | M | *Toxoplasma* | 18774 |
| 7BM | M | *Toxoplasma* | 71228 |
| 7CM | M | *Toxoplasma* | 50914 |
| 10AF | F | Placebo | 51330 |
| 10BF | F | Placebo | 45595 |
| 11AF | F | Placebo | 41060 |
| 12AF | F | Placebo | 58073 |
| 12BF | F | Placebo | 53514 |
| 2AF | F | Placebo | 19523 |
| 3AF | F | Placebo | 65877 |
| 3BF | F | Placebo | 73520 |
| 6AF | F | Placebo | 57325 |
| 6BF | F | Placebo | 62624 |
| 2AM | M | Placebo | 71065 |
| 2BM | M | Placebo | 63110 |
| 2CM | M | Placebo | 59530 |
| 4AM | M | Placebo | 57087 |
| 4BM | M | Placebo | 48666 |
| 4CM | M | Placebo | 58276 |
| 6AM | M | Placebo | 47834 |
| 6BM | M | Placebo | 22750 |
| 6CM | M | Placebo | 99413 |
| 8AM | M | Placebo | 64160 |
| 8BM | M | Placebo | 51330 |
| 8CM | M | Placebo | 45595 |

Table S2: Descriptive statistics of cleaned data for sequences per sample, summarised by experimental groups.

| **Treatment** | **Sex** | **Min.** | **1^st^ quartile** | **Median** | **Mean** | **3^rd^ quartile** | **Max.** | **n** |
| --- | --- | --- | --- | --- | --- | --- | --- | --- |
| *Toxoplasma* | F | 18165 | 49094 | 50824 | 49166 | 58119 | 66581 | 10 |
|  | M | 18774 | 51528 | 57534 | 52568 | 59997 | 71228 | 12 |
| Placebo | F | 19523 | 47028 | 54585 | 54325 | 63926 | 79103 | 10 |
|  | M | 22750 | 54981 | 58903 | 59320 | 63372 | 99413 | 12 |

Table S3: Differentially abundant bacterial ASVs in *Toxoplasma* infected rats relative to placebo controls (positive log fold changes represent higher ASV abundance in *Toxoplasma* infected rats, and *visa versa*).

*ASVs are labelled according to their closest available taxonomic classification. Data are based on 12 male and 10 female Toxoplasma treatment rats and 12 male and 10 female placebo treatment rats.*

| ASV-ID | Phylum | Family | Log fold change | Adjusted p-value* |
| --- | --- | --- | --- | --- |
| 14f0bc1d5dd538296342b5780d608154 | Bacteroidota | Muribaculaceae | -0.641159544 | 0 |
| 4d97b84bc44a0fe2c01b8827f87dc46a | Firmicutes | Lachnospiraceae | -1.025236578 | 0 |
| 3a3d386cd02b3285466fd0469a81b7c4 | Firmicutes | Lachnospiraceae | 1.176134416 | 0 |
| 0b98151fa888e12eee6c2a5ccc3797bf | Firmicutes | Lachnospiraceae | 2.040632705 | 0 |
| 366080a13899874610282fc1707bca2d | Firmicutes | Lachnospiraceae | -0.899719101 | 0 |
| 97db7e4bd47e0f424624f9bd8cf1c892 | Firmicutes | Lachnospiraceae | 0.570817769 | 0 |
| 6a0e701293d803c3b0622a7f556efbd6 | Firmicutes | Lactobacillaceae | 0.857066847 | 0 |
| f69416a5a3228fc61ecd4117a03dacd7 | Firmicutes | Lachnospiraceae | -0.366789559 | 0 |
| 2daab0076d4b922d0e6376717eda0ddf | Firmicutes | Lachnospiraceae | -0.4185757 | 0 |
| 4ec9d1c058be0d95620f23c41bd5fd16 | Firmicutes | Lachnospiraceae | -0.012443087 | 0 |
| 400058c29bc93490114416fe8b97df5b | Firmicutes | Oscillospiraceae | 1.290023344 | 0 |
| 1793b0e28d99f4322a9daaf67a04eecd | Firmicutes | [Eubacterium]_coprostanoligenes_group | 0.530037349 | 0 |
| 875d71019314210ee4249ee49680e3d8 | Firmicutes | Lactobacillaceae | 0.54539242 | 0 |
| 1391eb3de6a0651217250e4d62f4b6bc | Firmicutes | Lachnospiraceae | -1.126970804 | 0 |
| b00369f9fa4746a43df8527d95e066ce | Firmicutes | Lactobacillaceae | 0.625134497 | 0 |
| ccb0ae309eea797ca6bb97d76e7b2528 | Firmicutes | Oscillospiraceae | -0.638455826 | 0 |
| 09933ec3c71032907327154a75f7ff86 | Firmicutes | Lachnospiraceae | 0.459536855 | 0 |
| 51bd6c8fab51413e9b01260a2c7955d7 | Bacteroidota | Muribaculaceae | -0.456537417 | 0 |
| 7abd35bcc9c019b868db4c0388f8897a | Firmicutes | Ruminococcaceae | -0.528845079 | 0 |
| ebcda2225da7fa033a2f8379c08de7c0 | Bacteroidota | Bacteroidaceae | 1.086713619 | 0 |
| 42d17e34f2f8c4203d55d94ef05dd49f | Firmicutes | Lactobacillaceae | 0.448911389 | 0 |
| 47e5104819652573d9eabc2f06c353fc | Firmicutes | Lachnospiraceae | 0.255601093 | 0 |
| a4ef0fa99d2e5490753a86b078363554 | Firmicutes | Ruminococcaceae | -0.156211047 | 0 |
| 26cfc93699ea2930a69062043742217f | Firmicutes | Oscillospiraceae | -1.193423144 | 0 |
| c0f401c2ef7fd03f179ca1aa6e5a143f | Firmicutes | Lachnospiraceae | 0.425677327 | 0 |
| 77ad9b8e09f2a2ca59770bfa2304887c | Firmicutes | Lactobacillaceae | 0.558581984 | 0 |
| b3e17ca56ecbd13abdf870ccdf030dbd | Firmicutes | Lachnospiraceae | -0.391389711 | 0 |
| eb714857ee3ef9312860a7002237a767 | Firmicutes | Lachnospiraceae | 0.198389132 | 0 |
| a5a81a593f9852555bc08e6b779cf3d4 | Firmicutes | Lachnospiraceae | 0.47796859 | 0 |
| 110507e49a015e61e765d8aa331f2ff5 | Firmicutes | Oscillospiraceae | -0.670667574 | 0 |
| 5e5981acdb178ad1d8bcdcb6107e3606 | Firmicutes | Lachnospiraceae | 0.427492819 | 0 |
| 9abbfed5ecfb3f81fbd42308e9b84a60 | Firmicutes | Lachnospiraceae | -0.675582241 | 0 |
| c85f4e9bf0a8e439093aee34dc348ad8 | Firmicutes | Lachnospiraceae | 0.811946175 | 0 |
| c4440589bb9202601397be1c6b999240 | Firmicutes | Lachnospiraceae | 0.777432063 | 0 |
| ded961975d29e48b6ce7097df041d88b | Firmicutes | Lachnospiraceae | 0.267456456 | 0 |
| b7343d0e1a8702f311688616e258aa19 | Firmicutes | Lachnospiraceae | -0.47396465 | 0 |
| be6ab2f3768706e9b21d4a63462594fe | Firmicutes | Lachnospiraceae | -0.898227872 | 0 |
| 7df30163b4a34b9bc307fc35cdf73421 | Firmicutes | Lachnospiraceae | -0.476357772 | 0 |
| 68f1efc4e82d6386acfc9f18e02cbb8f | Firmicutes | Ruminococcaceae | -0.956580312 | 0 |
| 1ea04dbd20f5d46a1ce4f92084f4e5e5 | Bacteroidota | Muribaculaceae | 0.496958429 | 0 |
| 954fb3dc42ae6e607e043a54a4c683ed | Firmicutes | Peptococcaceae | -0.393231145 | 0 |
| 618026a71f6b73389dbafbf96cb3f779 | Firmicutes | Ruminococcaceae | 0.038160922 | 0 |
| 41319f55c576ac788eb7de20d46e89a6 | Bacteroidota | Muribaculaceae | -0.500865917 | 0 |
| fc951ae936cace2a5f6669556ea4d247 | Firmicutes | Lachnospiraceae | 0.271687143 | 0 |
| b47f9c8680b829a8f81043dd25c6e562 | Bacteroidota | Rikenellaceae | 0.102542129 | 0 |
| 0636ea6d334937b1f00b8a08be0b312c | Firmicutes | Lachnospiraceae | -0.202647104 | 0 |
| 177bb42e0bb13e0fd4a20f36c920da7e | Firmicutes | Clostridia_UCG-014 | 0.081864153 | 0 |
| b5622c29c9c003017947c332331501c0 | Firmicutes | Lachnospiraceae | -0.232596312 | 0 |
| 6a533037f2f22dc626ae5843b1294ab5 | Firmicutes | Clostridia_UCG-014 | 0.558990204 | 0 |
| 49051e3156aca0f509eda10f3b2e1447 | Firmicutes | Erysipelotrichaceae | 0.486946873 | 0 |
| dcbe129366ea512c75fa42d1a5a14aa4 | Firmicutes | Lachnospiraceae | 0.649967706 | 0 |
| 24576b11131854ded8a2de85c6ca7c49 | Firmicutes | Erysipelatoclostridiaceae | 0.128987654 | 0 |
| 4246ed701744ca6421467d73dc745333 | Firmicutes | Erysipelatoclostridiaceae | 0.080026068 | 0 |
| 2dc16e2e45d9433b606d8a2582c41e1d | Proteobacteria | Sutterellaceae | 0.286310692 | 0 |
| 899f472bcb20419a21a9af29154f02ea | Firmicutes | Listeriaceae | 0.974288949 | 0 |
| 4982bbaf199abfd1057d12302f1c1321 | Proteobacteria | Enterobacteriaceae | -0.456864662 | 0 |
| 86e98c8f3c07b2cff1ec6c535fc2e843 | Firmicutes | Lachnospiraceae | -0.465241367 | 0 |
| ad74db2c00fb7cb51430b8ed483b8d4e | Firmicutes | Lachnospiraceae | -0.254553858 | 0 |
| 2f862026b2c62871ab34f800583f6818 | Firmicutes | Ruminococcaceae | -0.763546741 | 0 |
| b8167fe5f74f5a7f5c51d24230d2b725 | Firmicutes | Clostridia_vadinBB60_group | -0.295886679 | 0 |
| f687c075625a3363bccac5afb14a2ea1 | Firmicutes | Clostridia_UCG-014 | -0.249849354 | 0 |
| 82aeb2674c4ee1359a1ae51302adf982 | Firmicutes | Lachnospiraceae | 0.695811245 | 0 |
| 5e5f0fe4d5ffc9ea017b9ab61e45a940 | Firmicutes | Lachnospiraceae | -0.185338156 | 0 |
| ac758289e53f97b02624c43428faec58 | Firmicutes | Oscillospiraceae | 0.252604968 | 0 |
| f1f7312902cbc9506cab3abeadf35fdd | Bacteroidota | Muribaculaceae | 0.357376386 | 0 |
| 9a6fa30bc30d7fbe45cf6a23fd75ec82 | Bacteroidota | Bacteroidaceae | 0.462885089 | 0 |
| cf50a3600632d6f3295fbd0ae6cf5d91 | Firmicutes | Lachnospiraceae | 0.15628711 | 0 |
| 3338e572407b529e052049ac657a45ae | Actinobacteriota | Eggerthellaceae | -0.130512328 | 0 |
| be2d5cd8bd3a6456cd9b030737e9e774 | Firmicutes | UCG-010 | -0.478789696 | 0 |
| 705225244c39e4aebfd6915bb2785b76 | Bacteroidota | Rikenellaceae | -0.252206808 | 0 |
| 7dd0503bd5d9fbe4a1109f490de31ea1 | Bacteroidota | Muribaculaceae | 0.221503728 | 0 |
| e6f1b967acef3cac744443b49362ad3f | Firmicutes | Clostridia_UCG-014 | 0.131300033 | 0 |
| cdcb548f94d85adad48983aacd53a195 | Firmicutes | Lachnospiraceae | -0.20318292 | 0 |
| 46fe23e81e0960b0c6f8372d3e7f7b4e | Firmicutes | Lachnospiraceae | 0.073124355 | 0 |
| 5dc5700c6b964caca60f3d99e5f7c29a | Firmicutes | Ruminococcaceae | -0.511080104 | 0 |
| 53df7425720628307e72fcd892ff75bc | Firmicutes | Lachnospiraceae | 0.326912416 | 0 |
| f2fa035b7abe124c51c534610a14a607 | Firmicutes | Lachnospiraceae | 0.087922069 | 0 |
| 91246a0dd8644b5af06f2e1efbe55b9a | Bacteroidota | Muribaculaceae | -0.270050521 | 0 |
| 49d07bea5874e5100baed9cbdbf7a0ba | Proteobacteria | uncultured | -0.125158554 | 0 |
| 3116337d58d37e192f7a5a57c274488d | Firmicutes | Oscillospiraceae | -0.494018689 | 0 |
| 20d4ecd56f68f21218683d6e4c13383c | Firmicutes | Clostridia_UCG-014 | -0.42217244 | 0 |
| 2245e8c1a37706a9cbe8c862be367ae6 | Firmicutes | Clostridia_vadinBB60_group | -0.147997526 | 0 |
| ed697f2adfae0581c01ef3785e43177c | Firmicutes | Lachnospiraceae | -0.274787658 | 0 |
| ee04964b969625477f819f8bac2a9271 | Firmicutes | Lachnospiraceae | -0.363419782 | 0 |
| 3c6d827c24ebaf7b2b96ec3f0114ea75 | Firmicutes | Clostridia_vadinBB60_group | -0.385119043 | 0 |
| 454d19277ec3b0c4e9be2b30ee5fdd13 | Firmicutes | Ruminococcaceae | 0.377915894 | 0 |
| 9fc4eec598ee512032ab2f17b7b36d33 | Firmicutes | Lachnospiraceae | -0.350948544 | 0 |
| 1cf852779a1fe6b8c5fd93a3a40163ea | Firmicutes | Oscillospiraceae | 0.168544545 | 0 |
| 1aa13180c75705a063351fec58e3a1fd | Firmicutes | Lachnospiraceae | 0.08628191 | 0 |
| 24c7c68810a247f369c580ef34ef9b4e | Firmicutes | Lachnospiraceae | 0.497241464 | 0 |
| 247445349af8eaaacbda7dc6d01bb9a3 | Firmicutes | Lachnospiraceae | -0.228670703 | 0 |
| 257226535a9992783b54605914b8e9ac | Firmicutes | Lachnospiraceae | 0.579540987 | 0 |
| 0978f5af3e0f5e47c8195884aea5a511 | Actinobacteriota | Eggerthellaceae | -0.173571718 | 0 |
| e18ebc11c35549dff2cad3d3b8d3a5b0 | Firmicutes | Oscillospiraceae | 0.06920863 | 0 |
| f1fdf96ec9180beddbcb9925b6ade1f1 | Firmicutes | Lachnospiraceae | -0.170222109 | 0 |
| f797b8fd39a4375220e49df334038081 | Firmicutes | Ruminococcaceae | -0.203093546 | 0 |
| 6e033c22f5d83d90e63c95dce55c471e | Firmicutes | Lachnospiraceae | -0.234470551 | 0 |
| a3f3a2084480e3d5779777fc0a9848aa | Firmicutes | Oscillospiraceae | 0.087370693 | 0 |
| 0963b7a69c0ce7467db9c0160afa4d33 | Bacteroidota | Muribaculaceae | -0.48529428 | 0 |
| e608db9a5b74b9cec1718486c80d041c | Firmicutes | Oscillospiraceae | 0.16782871 | 0 |
| 655d4560fd98bbd61b01d9ed02b07af2 | Firmicutes | Clostridia_vadinBB60_group | 0.205405009 | 0 |
| a747a614727beafaed3ec5a7b5ef4a51 | Firmicutes | Clostridia_UCG-014 | 0.345715228 | 0 |
| 338dea5b254da413f08f224dbc86d5cb | Firmicutes | Erysipelotrichaceae | -0.108946057 | 0 |
| 980a75ffc161ad8439740da5895aa8bd | Firmicutes | Lachnospiraceae | -0.086188182 | 0 |
| ada4dcf2ba628bde71d96a2df8332a38 | Firmicutes | Clostridia_vadinBB60_group | -0.199595317 | 0 |
| e4bdfbe01598ec48766d703391e453ea | Firmicutes | [Eubacterium]_coprostanoligenes_group | -0.451470933 | 0 |
| 553fb875c7866dc1f76b566c105586e1 | Firmicutes | Lachnospiraceae | -0.247535911 | 0 |
| 434df7f0bd3b837db50ebb809b44635a | Firmicutes | Lachnospiraceae | -0.3520582 | 0 |
| e8562100c942ba88e07cdf62fb680338 | Firmicutes | Clostridia_UCG-014 | 0.17040009 | 0 |
| 6166278e3f50703b5173c28c4727bb46 | Firmicutes | Ruminococcaceae | -0.05772864 | 0 |
| d9d008827daa2c1d5908184be9d67394 | Firmicutes | Lachnospiraceae | 0.208913629 | 0 |
| 60fd4278c813cbd214e74e18ca59d630 | Firmicutes | Clostridia_UCG-014 | 0.341139683 | 0 |
| 1d6f777ecfd47b7c578537a49025fa2a | Firmicutes | Clostridia_vadinBB60_group | 0.130149223 | 0 |
| 70ec22a41eff5dea8cb32c2b72744f9e | Firmicutes | Lachnospiraceae | 0.321407098 | 0 |
| 52f97d2d221f1a20416bfe6dd18289dc | Actinobacteriota | Bifidobacteriaceae | 0.343430203 | 0 |
| b52d2244952e405959ad253654273819 | Firmicutes | Peptococcaceae | -0.227146945 | 0 |
| d14eca8bec2e526a473a7a34fb6cb16a | Firmicutes | Ruminococcaceae | -0.206602166 | 0 |
| c3a9cc0f17fbabef7575115a82f991c6 | Firmicutes | Oscillospiraceae | -0.269615546 | 0 |
| 6e17ef4d2802807f06d1ad83321a6a1e | Bacteroidota | Muribaculaceae | -0.245115705 | 0 |
| 26fb1d3ebb5837c9cc111c418301cb74 | Firmicutes | Ruminococcaceae | -0.206602166 | 0 |
| c98fa8466f7ba0a709de9ec39cd81b9f | Firmicutes | Clostridia_UCG-014 | 0.092454707 | 0 |
| ec3491bf70ad4738b48db07f8a882115 | Bacteroidota | Bacteroidaceae | 0.205405009 | 0 |
| 6476b31c8ea466ad9c39095fb0db9c6d | Bacteroidota | Rikenellaceae | 0.160821861 | 0 |
| *Adjusted p-values for multiple testing using the Holm-Bonferroni method | | | | |
